# Transactions of the Medical Society of the State of New York

**Published:** 1850-07

**Authors:** 


Art. XI.—*Transactions of the Medical Society of the State of New York. During its Annual Session, held at Albany, February* 5, 1850.

We earnestly wish that the physicians of Kentucky could be induced to hold an annual convocation, similar to that held in New York. The proceedings of the Medical Society of New York, constitute a volume of great interest, and we are confident that the physicians of Kentucky could make a book equally as creditable and useful. But, we fear that there is an apathy among them that can scarcely be roused into activity. There have been two medical schools in Kentucky for many years, which should be some indication that the practitioners of the State are alive to the interests of their profession, but if they were called upon to point to the evidences of their devotion, we fear they would find themselves in difficulty. With an abundance of practitioners of skill, ability, and learning, Kentucky, may search among them in vain for anything like a general disposition to advance the interests of medical science; there are very few who are cultivating any one department of the profession with zeal and energy; and a very few who seem to care whether the cause of true science is in the ascendant or not. These things should not be so; there are numbers of medical men in Kentucky, who are capable of observing properly, and who can make the result of their observations, the common property of the profession. The pages of their medical journals do not, however, speak very creditably of their performances in the matter of writing. Where scores should write, there are scarcely half a dozen who do it. In other States, the physicians act upon the principle that the medical journals of the State are mirrors which reflect the character of the profession, and they do all in their power, to make the image reflected, respectable, honoring, and ennobling. And why should the physicians of Kentucky be less alive to their character, than those of other States? We hope they will prepare themselves for showing that they can redeem the time that has been lost, and can do noble service for a noble cause. This number is the first of a new volume of the Western Journal of Medicine and Surgery, and the physicians of Kentucky should show the world what they can make of a Western Medical Journal. We are well pursuaded of what they can do if they try, and we wish them to try.

In addition to their labors upon the pages of the medical journals printed in the State, we think it is the bounden duty of the medical men of Kentucky to establish a State Medical Society, in which they can meet once a year, and mingle together for the benefit of medical science. In this way they would advance the entire interests of the profession; while improving themselves, they would contribute powerfully to the success of the National Association of Physicians; by becoming personally acquainted, and renewing the acquaintance annually, they would gradually form the links of a chain that would encircle the entire interests of the profession, and would push its usefulness, honor, and worth, upwards and onward. It should not require much argument, nor much appeal to bring about this state of things in Kentucky, and we submit the subject to the consideration of the medical men of the State.

In this appeal to the physicians of Kentucky, we have almost lost sight of the transactions of the Medical Society of the State of New York. There are several papers, in the volume, of great value, and we take peculiar pleasure in calling the attention of our readers to them.

The first paper is the annual address, before the Medical Society of the State of New York, and members of the Legislature, by Alexander H. Stevens, M. D., president.

The theme selected by Dr. Stevens, is one of the most important that can engage the attention of a civilized people, and in the ratio of attention to the subject is the estimate we must make of their civilization. We allude to Hygienic measures for the public health. The facts on this subject cannot be too often presented, as long as there is any neglect by the public authorities. There are two things upon which all governments should be founded; one is that it is the duty of the State to make ample provision for the education of every child within her territory; the other is, that it is the business of the State to remove all causes of accidental disease. These are paramount duties and can never be safely neglected. As long as the taxes collected from the people are invested in these two objects, they are in profitable stocks that yield the largest and best of dividends. Statesmen talk about the duty of the government to protect citizens in life, liberty, and property, but what is that protection worth, that neglects education and health? The deaths in the city of New York, last year, amounted to twenty-two thousand three hundred and seventy-three—of this large number, only two hundred and twenty-four died of old age, of the remaining twenty-two thousand one hundred and forty-nine, an immense proportion died from causes that sanitary regulations would have removed. Where then was the protection in life, liberty, and property afforded by the city authorities of New York in the year 1849, when the civilized world is blazing with light, on Hygienic measures? What if a mayor, city attorney, policemen, and all the immense machinery that moves city laws, were paid from the taxes of the people, while the gates of death were permitted to close upon twenty-two thousand one hundred and forty-nine beings, who might have been saved? If the mortality was great and appalling what must we say of the sickness, that was not fatal? A fair estimate places this at two hundred and twenty thousand cases, a large proportion of which should have been avoided. But governments are supposed to perform their duties, if they arrest house breakers and thieves and send them to the penitentiary; if they hang murderers, and send children to the work-house, to be educated in crime, so that the State may graduate them, hereafter, in the penitentiary. The world is truly said to be governed by names; political questions set the community into a blaze, while all that is really important to the interests of humanity is lost sight of. Lawyers guide the public mind into the worn ruts of professional interests, while the schoolmaster and the physician look on the scene as if stricken with palsy.

The physician should awaken to his duty. His is the highest of all earthly missions, and no one should seek admission into the temples of Esculapius, who is not prepared to devote himself to the public welfare. The activities of the age are around the physician, and he must not slumber at his post. The long night of selfishness, which has reigned upon the earth, which has strewed the pathways of the world with human bones, and dyed the fairest fields of the earth with blood, is breaking up, and the dawn of a brighter day is revealing itself. The great principle that it is necessary to take care only of number one is beginning to disappear, and we find men everywhere trying to recognize the truths, that humanitarianism is abroad, and that he who does the most good to the greatest number is the only true man. In these high and ennobling pursuits, the physician must take his place in the van, and teach the great truths of Hygienic science to the world. This has ever been the guiding star of medical men in all ages; they have ever been philanthropists in word and deed, but never until now, has the world been attentive hearers of their lessons. A great work is in progress, and medical science must light up the investigation with all that will enable men to see the subject properly.

We boast great cities in the West and South. They are the magic creations of the energies of a wonderful people, and we should rejoice to see that while these cities grow in greatness, they also grow strong in all things that tend to give health to body and mind. Cincinnati, Louisville, St. Louis, and New Orleans are already great among the cities of the Union, but they have not yet advanced so far that they cannot adopt a system of measures that will protect the health of the public, and to that great end all measures should look. The cities we have named are yet in their infancy, and should consider themselves but children at school, under the tuition of the older cities of the earth. A long, bitter, painful, and losing experience has taught London, Manchester, Leeds, Edinburgh, Dublin, and many such cities, the absolute and essential necessity of attention to sanitary measures, and the younger cities of the New World should rather profit by the dear-bought experiences of others, than wait to feel them in their VITAL STATISTICS. It is in the power of medical men greatly to diminish the evils that afflict cities, and they should consider themselves soldiers enlisted for the war, and never sheath their swords, nor ground their arms, as long as aught remains to conquer from the remediable causes of disease. The public mind should be kept awake; its unceasing energies should be directed to causing a full and faithful attention to all sanitary measures.

In the address of Dr. Stevens, which has called forth these remarks, a number of things are mentioned as "prominent evils of our social condition in relation to public health," and we quote, what we consider the well ascertained causes of disease. General sickness arises "from the imperfect drainage of surface water, of spring water and of impurities. From ignorance or the want of proper attention to the means of rendering dwellings healthful in the heating, lighting, and ventilating of them. From the construction of mills, canals, railroads, &c., in such manner as to arrest the natural course of water towards the ocean. From unhealthful manufacturing establishments and occupations, and from improper burial of the dead. From the want of due ventilation of yards, low grounds and confined places out of doors. And lastly, from the want of a more complete protection against small pox by enforced vaccination. What remains of the sanitary art belongs to medical men individually."

To these points we consider it the duty of medical men in cities to turn their attention. The cities of the West and South should not be permitted to grow in a wild and reckless disregard of everything that tends to protect the people from sickness. It may not be in the power of physicians nor of municipal authorities to open up mines of wealth in the abodes of poverty, but it is within the power of men to give sunshine, and health-giving air to the humblest retreats of the poor. The life of the poorest man in the community is as valuable to him, and to those who lean upon him, as the life of the millionaire is to him, and should be equally as much a matter of public concern, at least as far as sanitary measures may be protective. There is no excuse for neglect of these things. It is not necessary that the poor shall breathe an air tainted with death; that a dark cloud shall forever hang around them, in the workshop, and in the hovel. It is not essential to the good of society that the afflictions of sickness shall be superadded to the embittering evils of poverty. If the municipal authorities have not the right to forbid the erection of houses that are unfit for the preservation of human life, why are they empowered to forbid the construction of powder magazines? Municipal authority claims the power to forbid the erection of frame buildings in particular localities, lest fire should endanger the neighborhood. Should it not be equally as guarded against pestilence? Should it not be equally as prompt to prohibit a man from arranging his house or yard in such a way as to be injurious to the health of the occupants, or of the neighbors? Those who build dens of pestilence for the poor have a terrible account to render some day, and a day of reckoning will surely come. These things should be looked into, and city authorities should have the right to say that no man shall build a dwelling house that is not thoroughly ventilated, not only in the rooms, but from cellar, or ground floor, to the attic. Thorough drainage should be enforced in all cases, and attention should be demanded for all those means of health, that experience has discovered. Such buildings cost but little more than the things that are called cheap houses, and the landlord derives the benefit of attention to the health of his tenants, in their improved means to pay his demands. A poor family often loses more than the amount of rent in sickness that wastes their means, stops their labor, and robs them of employment. And can any one undertake to say that these are not proper objects of a city's care? Can there be anything more important in regulating the sources of prosperity than that which looks to the health of the laboring poor? We speak of the poor, because the rich generally take care of themselves, but the rich are interested in the matter we are endeavoring to urge. There is not a being on the broad earth who is not interested in it. It is linked indissolubly with the weal or woe of the human race; it presses daily and nightly upon the senses of men, and none can stifle the conscience so far as to shut out the truth that society must look into its foundations, or the superstructure will fall.

The most eminent, by far the most vital of the novelists of the day, has drawn a picture so much in keeping with what we are trying to impress upon the minds of our readers, that we cannot do justice to the subject without permitting Mr. Dickens to display those Salvator Rosa hues, of which he is a thorough master. There are few city physicians who may look on this picture, without feeling that even the rich imagination of Dickens has failed to picture forth all the horrid realities that meet the eye of the practitioner of medicine.

Mr. Dickens thus discourses: "But follow the good clergyman or doctor, who, with his life imperilled at every breath he draws, goes down into their dens, lying within the echoes of our carriage-wheels and daily tread upon the pavement stones. Look round upon the world of odious sights—millions of immortal creatures have no other world on earth—at the lightest mention of which humanity revolts, and dainty delicacy living in the next street, stops her ears, and lisps 'I don't believe it!' Breathe the polluted air, foul with every impurity that is poisonous to health and life; and have every sense, conferred upon our race for its delight and happiness, offended, sickened and disgusted, and made a channel by which misery and death alone can enter. Vainly attempt to think of any simple plant, or flower, or wholesome weed, that, set in this fetid bed, could have its natural growth, or put its little leaves forth to the sun as God designed it. And then, calling up some ghastly child, with stunted form and wicked face, hold forth on its unnatural sinfulness, and lament its being, so early, far away from Heaven—but think a little of its having been conceived, and born, and bred, in Hell!

"Those who study the physical sciences, and bring them to bear upon the health of Man, tell us that if the noxious particles that rise from vitiated air, were palpable to the sight, we should see them lowering in a dense black cloud above such haunts, and rolling slowly on to corrupt the better portions of a town. But if the moral pestilence that rises with them, and, in the eternal laws of outraged Nature, is inseparable from them, could be made discernible too, how terrible the revelation! Then should we see depravity, impiety, drunkenness, theft, murder, and a long train of nameless sins against the natural affections and repulsions of mankind, overhanging the devoted spots, and creeping on, to blight the innocent and spread contagion among the pure. Then should we see how the same poisoned fountains that flow into our hospitals and lazar-houses, inundate the jails, and make the convict-ships swim deep, and roll across the seas, and over-run vast continents with crime. Then should we stand appalled to know, that where we generate disease to strike our children down and entail itself on unborn generations, there also we breed, by the same certain process, infancy that knows no innocence, youth without modesty or shame, maturity that is mature in nothing but in suffering and guilt, blasted old age that is a scandal on the form we bear. Unnatural humanity! When we shall gather grapes from thorns, and figs from thistles; when fields of grain shall spring up from the offal in the byways of our wicked cities, and roses bloom in the fat churchyards that they cherish; then we may look for natural humanity, and find it growing from such seed.

"Oh for a good spirit who would take the house-tops off, with a more potent and benignant hand than the lame demon in the tale, and show a Christian people what dark shapes issue from amidst their homes, to swell the retinue of the Destroying Angel as he moves forth among them! For only one night's view of the pale phantoms rising from the scenes of our too-long neglect; and, from the thick and sullen air where Vice and Fever propagate together, raining the tremendous social retributions which are ever pouring down, and ever coming thicker! Bright and blest the morning that should rise on such a night: for men, delayed no more by stumbling-blocks of their own making, which are but specks of dust upon the path between them and eternity, would then apply themselves, like creatures of one common origin, owing one duty to the Father of one family, and tending to one common end, to make the world a better place."

The good spirit that is to "take off the house-tops with a more potent and benignant hand than the lame demon" of Le Sage is the medical profession. Upon that profession the duty must devolve; through them, humanity, in all its grades, must enjoy the life-giving energies that belong to the earth; through them the hovels of wretchedness must be emancipated from all those ills that speak so plainly in the condition of Mrs. Pipchin's school house, in which "air could not get out in winter, nor come in, in summer."

We heard of a case sometime ago, that occurred in Meade county, which forcibly illustrates much of what we have just been urging. In the range of the practice of Dr. Young, an eminent physician of Hardin county, in the adjoining county of Meade, there were two families who lived within four miles of each other. Each of these families was attacked with a violent form of dysentery, while the neighbors were generally healthy. Dr. Young, who considered that a physician has other duties to perform, besides administering medicine, endeavored to ascertain the cause of this dysentery. He could find nothing around the house, nor any improprieties in diet, that satisfied him as to the cause, but having resolved to find a solution to the mystery, he persevered. He was struck, at length, by the fact that the underpart of the frame, or hewed-log houses, was not ventilated, and upon knocking holes through the foundation walls, he discovered the object of his search. Pools of water were standing under the floor affording moisture to the decaying vegetation that was on the surface of the ground. These pools were made by repeated washings of the floors, which were not made of tongue and grooved plank, and, of course leaked. The immediate effect of Dr. Young's extemporaneous ventilation was the abatement of the disease, and the two families recovered rapidly. Had it not been for his persevering investigations into the cause of the sickness of these families, the strong probability is that there would have been several deaths in the two families. But in a milder view of the cases, these families were saved from long continued suffering, loss of time and of productive labor, shattered constitutions, and those expenses that are incident to sickness. It is for considerations of this kind, that we appeal to the State authorities to interpose everywhere, within their jurisdiction, in order to save the people from suffering. Of the extent of the losses in some of the items we have named, few have any just conception, but we shall endeavor to point out these matters in a subsequent part of this article.

We believe that what Dr. Young did on a small scale in Meade county, the State authorities could do throughout the State. For the adjudication of miserable little debts, from a dollar up to fifty, every neighborhood in the State is provided with an expensive little court, but in no part of the State is the least care manifested in the more important matter of health. Mill-dams are erected without the slightest regard to the immense injury that may be done to lands, heretofore, drained by the creek that is dammed, and to families protected, hitherto, from sickness, by the drainage. People, in ignorance of such things, build dwelling houses on the most pestilential portions of their lands, and often destroy a belt of timber or shrubbery that protects them from the contiguous marsh. These, and hundreds of kindred things deserve the attention of the State authorities, and the people should see that they receive attention. The establishment of a sanitary board in every part of the State, with ample powers for the removal of all known causes of sickness, would render the Commonwealth more service than she has ever derived from her county courts, but we wish to be understood as not attempting to depreciate their value. But we hold it to be an undeniable truth, that the public authorities should preserve the people from all removable causes of disease, and that this is a paramount duty.

The following case, referred to by Dr. Stevens, illustrates what we meant in speaking of mill-dams. Dr. Stevens says: "A volume might be filled with proofs and illustrations of the shortening of human life, and the unfitting of men for labor by a want of due knowledge on the one hand, and of due legislation on the other. Even in Massachusetts, where marsh fevers are not so rife as with us, or so readily induced by stagnant water, the canal near Springfield caused so much sickness that it was indicted, presented by a grand jury, and its location changed in consequence."

Here is another important subject: "Now let us look at other sources of disease. According to M. Dumas, a man changes into carbonic acid in one hour, all the oxygen contained in ninety litres (about 3 1-6 cubic feet) of air, and the volume of all the air expired, which is about 13 cubic feet, contains about 4 per cent of carbonic acid. The quantity of heat disengaged, is equal to that arising from the burning of as much charcoal as the blood loses of carbon. Thus every breathing individual is a small furnace, fed by the air, and discharging its smoke into the room. Now suppose two thousand such living furnaces collected, as I have recently known in the city of New-York, in a newly built church, imperfectly ventilated, each burning nearly ¼ of an ounce of charcoal every hour, 1,000 ounces, or 83 lbs., during a two hours service; and this in addition to the admixture in the same atmosphere of sulphuretted hydrogen ammonia from the surface of the body, and the further deterioration of the air by gas light. Is it to be wondered at that faintings and permanent sickness should follow such assemblies? The common mode of suicide in Paris, is by breathing the vapor of burning charcoal. The burning the carbon of our bodies and breathing its noxious gases in close and ill ventilated apartments, is only a slower suicide."

On the subject of vitiated air in workshops, Dr. Stevens says: "Among literary and professional men, sedentary pursuits do not in England, says Professor Guy, shorten life. 'There is no other cause to be assigned for the great prevalence of consumption among those who labor in workshops, than vitiated air.'

"The Register-General reports nearly 60,000 deaths from consumption every year, in England and Wales. Professor Guy thinks 36 000 only of these are true consumption, of which he believes 5,000 might be annually saved, and half the number in London. One-sixth of the whole are among the laborers; he attributes these to the foul air of the work shops, the remainder to the condition of the dwellings of the poor. Deficient ventilation produces more consumption than all the other causes put together. Intemperance is the next greatest cause of the shortening of human life. 'The unwholesome state of the air in the work shops, especially printing offices and tailor's shops is the great cause of intemperance among the workmen.'

*Dr. Guy gives the following table, illustrating the influence of want of ventilation among letter press printers.*

| | Spitting of blood. | Catarrh. | Other diseases. | Total. | Per centage proportion. Spitting of blood. | Catarrh | Other dieea-ses. | Total. |
|---|---|---|---|---|---|---|---|---|
| 104 men, having less than 500 cubic feet of air to breathe.............................. | 13 | 13 | 18 | 44 | 12.50 | 12.50 | 17.31 | 42.31 |
| 115 men, having 500 to 600 cubic feet of air to breathe................................ | 5 | 4 | 23 | 32 | 4.35 | 3.48 | 20.00 | 27.82 |
| 101 men, having more than 600 cubic feet of air to breathe.............................. | 4 | 2 | 18 | 24 | 3.96 | 1.98 | 17.82 | 23.76 |

"Sir James Clark, who has written the best work on consumption in the English language, says, the respiration of a deteriorated atmosphere is one of the most powerful causes of tuberculous cachexia, the medical term for the state that precedes consumption. He says, 'if an infant born in perfect health, and of the healthiest parents be kept in close rooms, in which free ventilation and cleanliness are neglected, a few months will often suffice to induce tuberculous cachexia. Children raised in workhouses and similar establishments almost always become scrofulous, more from bad air, than defective nourishment.'

"Mr. Toynbe testifies before the sanitary commission that the poor who had ventilators introduced in their rooms say, 'they are more comfortable and airy,' 'that smells are no longer perceived,' 'they are more grateful for the ventilators than for milk and flannel.' *First Sanitary Report, p.* 337.

"In the Norwood school were six hundred pupils. Scrofula broke out extensively among them. It was attributed to bad and insufficient food. Dr. Arnott was consulted. He attributed it to foul air. Ventilation was introduced, and the scrofula disappeared. Eleven hundred are now maintained in good health, where before six hundred were sickly."

On the political economy of this subject, Dr. Stevens arrays a number of striking facts. We quote from him:

"Lord Morpeth remarked in Parliament in April, 1847, that in the various large towns in England, there were above 70,000 cases of unnecessary sickness, and in London about 250,000 such cases, and 10,000 deaths annually that might have been prevented. 'There are other items of expense,' he very justly observes, 'such as direct attendance on the sick, the loss of their labor, the premature death of productive contributors to the national wealth and the expense of premature funerals.'

"What the total amount of unnecessary, (and let me add as I believe, increasing) mortality in this State is, we have no precise means of knowing. In England it is estimated that of 300,000 deaths, only 35,000 are from the decay of nature.

"Dr. Southwood Smith calculates the annual slaughter in England and Wales from preventive causes of typhus alone, among persons in the vigor of life, at double the amount that was suffered by the allied armies at the battle of Waterloo. In our Mexican war, among the volunteers, 'the proportion of those cut down by sickness to those who fell in battle is as five to one.'* They had not in all cases the services of surgeons as well educated as the army surgeons.

"Dr. Playfair estimates the loss for the town of Manchester at one million of pounds sterling; that of London at two millions and a half; that for England and Wales little short of eleven millions; and that of the United Kingdom at twenty millions of pounds sterling every year. In some of the manufacturing towns in Great Britain, the number of children who die before they reach the age of productive usefulness, and who may be said to die indebted to the State, is greater than the productive wealth of their parents. In the State of New York life

*See Gen. Taylor's address to the volunteers.

and labor are of far greater value than in England, and our malarious and undrained country is far more unhealthy. In the cities of New York and Boston the average of human life is very considerably shorter than in London. In Massachusetts it has been ascertained that the average period of life of those who survived the age of fifteen years, was thirty-nine years and seven months nearly. In this State, suppose the average duration of life to be 39 years. The productive period begins at fifteen, and allowing four years to pay the debts of childhood, there are left only twenty productive years. Every year therefore added to human life increases the productive wealth of the State nearly 5 per cent.

"I am indebted to Archibald Russel, Esq., for the following reference to Carey's Political Economy, Part II., p. 285: 'In estimating the average production at $95 per head, or giving $380 for each family of four persons, we believe *we shall not vary materially from the truth.*'

"Population of the State of New York in 1840, 2,428,-921.

"Estimated population of the State of New York in 1850, 3,200,000, which according to Carey at $95, will be $304,000,000. This is the gross sum of production, i. e., of production beyond waste, loss and consumption. Five per cent on this sum is the real amount added to the productive power of the State by the lengthening of life one year, and is equal to $15,200,000.

"This loss is from premature death; but death and sickness go together. It is estimated that for every death there are two years or seven hundred and thirty days of sickness and of lost labor, leaving out of view the expenses of attendance, the entire derangement in the course of labor in a whole family, which the sickness of one of its members occasions, there is a direct loss probably at least equal to that from death. In all, more than $30,000,000.

"The fact to which I would now call your attention is, that the causes of a large part of diseases are to a very great extent proved to be removable. The chief means, so far as the public is concerned, are drainage, cleanliness, the due warming and lighting of our apartments, the ventilation of them and our grounds. 'The subject of the preservation of health,' says Dr. Neil Arnott, 'is rendered remarkably simple by the fact that in regard to all things over which we can exercise control, there are only four which man requires, air, temperature, aliment and exercise, and two which he should avoid, poisons and violence.' To these I would add a quiet conscience and well regulated mind."

Queen Victoria in her speech from the throne, at the opening of the present session of Parliament, thus expressed herself: "Almighty God in his mercy has been pleased to arrest the progress of mortality, and to stay the fearful pestilence. Her Majesty is persuaded that we shall best evince our gratitude by vigilant precautions against the more obvious causes of sickness, and an enlightened consideration for those who are most exposed to its attacks." If she could thus talk of the poor and suffering, and invoke the care of Parliament for them, might not a Governor of Kentucqy, call upon the Legislators of the State to awaken to the important duty of preserving the public health? The remark of Dr. Stevens on this point, for the State of New York, should become a tenant of the mind of every man in Kentucky:

"Whatever plan legislative wisdom may adopt, this fundamental fact must be recognised, viz: that a scientific and medical element has become essential to the due organization and administration of your State government. The importance of the ends in view, the magnitude of the means required, render it idle to trust to impromtu advice and opinions."

We cannot quit the important subject, without calling attention to the important facts contained in the appendix to Dr. Stevens's address. These facts are connected with the sanitary construction of country dwellings, ventilation, drainage, &c., and bear on subjects on which medical men are often questioned, and on which they should be prepared to give correct information. We present a synopsis of the valuable views given in this appendix; they are in accordance with the time-honored observations of the fathers of medicine, and their successors, and many of these facts were well known to the classical writers of Greece and Rome.

1. The site for a country dwelling should be chosen in reference to its height above the adjacent ground, its exposure, its drainage, and the quality of its water. Springy grounds should be avoided. If there is a marsh, or any other source of malaria, the house should be to the westward of it, because westerly winds are most prevalent. In all countries the winds are the purifiers of the air. If there is a belt of wood between the house and the swamp, it shonld be left as a protection against malaria. If there is none, a screen of pine or other dense growing trees should be planted, such trees as will attain a height of at least twenty feet. (In the absence of evergreens, we consider the *Pawlonia Imperialis* the best tree for this purpose. It will grow from twelve to fifteen feet in a season, if it has a good soil; its limbs are compact, the leaves very large, and it is one of the most beautiful of all flowering trees.—*Editor West. Jour. Med. and Surg.*) If there is any reason why water should gather around the foundations of the house, a drain should be cut so as to prevent it. Dry foundations prove the dryness of the contiguous grounds. A well near a house affords a good drainage; the deeper it is the better. Trees should not be left too near the house, nor should rank herbage be permitted to grow and decay in the immediate vicinity.

2. When there are no sufficient reasons for another preference, a south-easterly aspect is the most healthful for a residence. Trees on high land on the north-west are a protection in winter. Whatever the aspect of the house, the south side should have its full share of windows. Southerly windows are the most pleasant in winter, and are unobjectionable at all other seasons.

3. The cellar should be dry, clean, well ventilated, and well lighted. No wood, nor decaying vegetable matter should be placed about the cellar. It should communicate with the air above the house by a flue in the chimney stack. In default of this, it should be ventilated by lateral apertures. If any part of the cellar is floored, the air should be drawn from beneath the floor into a chimney flue. Chimney flues should be round—for bituminous coal, Tredgold says the diameter of the flue in inches should be equal to the square root of the height in feet: thus 50 feet high would require (7+7=49) seven inches.

4. Every bed room, especially if small and without a chimney flue, should have a ventilating aperture not less than four inches square, communicating with the attic above, and led through the roof by a proper conductor, except in cases where the door of another room can be left open; in this case the ventilation is sufficient. No bed room should be on the ground floor. But rooms on the second floor are healthful, those in the attic still more so. (Universal observation proves the truth of these views.—*Ed. West. Journ. Med. and Surg.*)

5. Piazzas, used as sitting places in summer and autumnal evenings, except those on the southerly or westerly sides of houses built with stone or brick are unhealthful. Stone or brick walls retain the heat of the afternoon sun, until a late hour at night. A veranda with windows is not liable to the same objection. It is essential that a house shall be well lighted by the sun.

6. The windows and chimney of a room represent a syphon, with one branch perpendicular and the other horizontal. The air should enter at the doors and windows, and rise in the flue. Dr. Arnott, of England, has invented a self-acting valve, which prevents a back current.

7. A fire in damp weather, during autumnal evenings, and even in the cool evenings of summer, is exceedingly healthful. If there is absolute necessity for a lower bedroom, a fire in the early part of the evening should always be made during the sickly season. It ventilates the room, brings in fresh air, and dries the air thus introduced. *Dry air is not a vehicle for malarious emanations.*

8. A sitting room should be well lighted by the sun's rays.

9. A tallow candle deteriorates the air rapidly, and when not snuffed, the deterioration is still greater. Gas is less vitiating. The burning of a tallow candle raises in one hour the temperature of twenty-seven meters of air, from the freezing to the boiling point of water. A carcel lamp in one hour raises from the freezing to the boiling point, fifty cubic meters of air. These facts will enable any one to judge of the sources of heat and vitiation in crowded and well-lighted apartments.

10. Collections of persons in a room, vitiate the air by emanations from the bodies, and by respiration. Ten cubic feet per minute is the amount necessary for the healthful respiration of one person. This should be doubled for bed rooms, and fourfolded for sick rooms. Irregular draughts of air may be prevented by making the air enter the room through wire, cotton, or silk gauze.

11. Upper apartments are safest against malarious diseases, but most dangerous for diseases arising from emanations from the bodies of the sick.

12. The dangers of low, undrained lands, and of fogs from such localities are well known. Drainage removes these dangers. It has been found that drainage in England has elevated the temperature on some occasions six degrees, and evening chills are no longer experienced in welldrained localities. In New York the temperature is said to have been raised 15° by drainage. Thus comparative immunity is afforded not against marsh fevers only, but cholera, rheumatism and acute inflammatory diseases. "In the statistical account of Scotland are found among many others the following notices of the great advantages derived from draining. In *Fourdown*, 'so much drainage that now no sickness; formerly agues common, now quite unknown;' in *Carmylie*, 'health improved from drainage, Kènnon agues very prevalent sixty years ago, now never met with,' and a long chapter is filled with similar statements from all the rural districts of England and Scotland. But why look abroad. In Onondaga valley, New York, health is much improved by its drainage and the experience of every one will supply facts in illustration.

"It is not sufficient to dry only the surface of the ground by drainage, in order to prevent effectually the formation of miasmata; it is essential that the drainage should be deep and thorough, for the poison may emanate from moist decaying vegetable matter below."

13. The modern art of engineering employs new and cheaper means than were formerly used for draining. I allude to the substitution of small pipes for larger drains, from the discovery of the fact that they are less liable to become obstructed, provided they have a descent of not less than 1 per cent of their length.

"Now it is proved that whilst house drains of such sizes and construction as have been enforced by the commissioners of sewers, accumulate deposit, drains of a much smaller size keep perfectly clear. Thus whilst a twelve inch drain, which is required by the Kent and Surrey, and the tower hamlets, and the city commissioners, accumulates deposits and generates noxious gases, a tubular earthenware drain, of nine times less capacity, or of four inches in diameter, or proportional to the house of from three to six inches, keeps perfectly clear. Even three inch drains convey away the refuse from middle-sized houses, and keep perfectly clear, whilst the larger permeable brick drains, which are usually charged three times the price, are choked up."

Mr. Roe, the surveyor of the Holborn and Finsbury district of sewers, who led the way in systematic improvements in the form and construction of main lines of sewers in the metropolis, recently, at our suggestion, made experiments on the rate of flow of water through the common brick drains for houses, as compared with the rate of discharge through earthenware drains of the same capacity, and with the same run of water. The general results which he gives are, that through the earthenware tubes the rates of discharge are increased to an important extent—in the smaller and more frequent forms, to the extent of more than a third. In other words, an economy of one-third the quantity to obtain the same result is effected by them, and the general efficiency of the drainage in ordinary runs proportionately augmented, as will appear, at a greatly reduced price.

"The following are examples:

*Table of the comparative run of water through brick drains and glazed pipes.*

| Inclination. | Depth of water | Time through glazed pipes. | Time through brick drain. |
|---|---|---|---|
| Level............. | 5 inches | 38 | 50 |
| 2 inches in 50 feet, | 4½ " | 16½ | 25 |
| 1¾ " " | 5½ " | 19 | 27 |
| 2¼ " " | 3 " | 18 | 26 |
| 1½ " " | 3½ " | 25 | 36 |
| 3¼ " " | 4 " | 15 | 22 |
| 2¾ " " | 6 " | 13½ | 21½" |

*First Report of Metropolitan Sanitary Commissioners.*

Mr. Chadwick, of London, has received from Switzerland a pipe which has served for drainage 500 years without injury or obstruction, under the pressure of a great head of water.

These are facts of great value, and should be well and strongly impressed upon the public mind. We have been intending to prepare a set of rules on these subjects, but Dr. Stevens's suit our views so well that we prefer using his. There is but one defect in them, and that is the absence of all reference to filth and uncleanness. Families that have dirtier habits than a free hog, and there are many such, may attend to all the thirteen rules given above, and still be sickly. If there is any one vice for which society should extend no pardoning gifts, it is the wickedness of uncleanness. Those guilty of it should have no vernal bloom, nor autumnal fruits to bless their cheerless filth.

A Sonth Carolina writer has recently published an interesting article on the subject of malaria, which has been extensively quoted. This shows the interest felt in the subject. We earnestly wish the gentleman had been more familiar with his subject, for his sins of omission were many, and of commission not a few. That article, however, was based upon the writer's knowledge of the immense losses in the South, that are consequent upon ignorance of the proper mode of managing malaria. *From the examination we have given the subject, we do not entertain a doubt, that the annual losses, from sickness in the United States, from causes that could be easily and cheaply remedied, amount to a sum sufficient to erect commodious school houses at convenient distances all over the United States, and to defray the expenses of giving a good education to every child in the Union. Is it not time then that somebody was moving in this momentous matter?*

We have dwelt upon this subject because of its vast importance, and after all we have said, we know that we leave the subject unexhausted in many particulars. He that improves the public health improves the whole machinery of society, for without good health, there is neither prosperity, nor morality. A healthful turn of mind is the proper condition for the cultivation of the moral and religious principle. The hand that scatters plenty o'er a smiling land, and the arm that protects a community from the storms of war, are considered worthy of especial honor among men, but how much more worthy is he who checks the devouring pestilence, stays the fiery plague, and removes from the hovel and the palace, the cellar and the attic all those insidious foes that undermine human happiness, health, and life? What holier, higher duty can be performed than in visiting the poor and wretched with the ministrations of health and comfort; than in showing them that in their warfare with the ills of poverty, the eye of beneficent power is over them to guard their nightly slumbers from poisonous atmosphere; to cheer their toils, and the domestic hearth with health and peace, and to crown their lives with immunity from all sickness that springs from the bosom of the earth. If a commission were appointed to gather the VITAL STATISTICS of Kentucky, and the duty were performed with fidelity, there would be an appalling picture presented to the eyes of the constituted authorities of the State. Every district, every neighborhood would teem with facts and figures showing everywhere duty unrecognized, and duty unperformed. The disproportion between those who die of old age, and those who die from avoidable causes of sickness would cry aloud against that neglect that leaves the citizen a victim to ignorance and the violation of all those laws that promote health and vigor, and that clothes families with misery, as with a garment, robs society of a member, and the State of what should be an object of its solicitude and care.

The time will come when the State authorities will not be permitted thus to trifle with the highest interests of humanity, and medical men should hasten the hour of its approach. This is the kind of medical politics to which physicians should devote themselves. We have merely suggested the matter, but we do not intend to permit it to rest.

Once more we appeal to the medical men of Kentucky to turn their attention to the subject of public hygiene. District and county medical societies should be formed throughout the State, and a State Medical Association should grow up from these elements. The public authorities and their master—the people should be enlightened by the press, and by oral eloquence, until the State shall repose in security under THE EGIS OF THE PERFECTION OF SANITARY MEASURES.

The important subject of public hygiene is ably handled by Dr. Stevens, and we cordially thank him for bringing it before the State Medical Society and the legislators of New York.

There are several other papers in these proceedings that deserve a synopsis, but we have not space to spare at present, for anything more than a passing reference to them.

A valuable paper, entitled: "Contributions to the Vital Statistics of the State of New York, by Lemuel Shattuck, Esq., was communicated by T. Romeyn Beck, M. D.

The paper on Hygiene and Medical Statistics, by Dr. Charles A. Lee, contains a number of interesting facts on the subject.

We are pleased to learn that the late Dr. Forry, of New York, left a manuscript work, entitled: "Vital Statistics, the Development of Man's Faculties, and the Laws of his Mortality and Reproduction, viewed in the relations to Hygiology, or State Medicine." All who know the eminent abilities of Dr. Forry, will hail with delight, the publication of his work.

The address of Dr. Swinburne, vice president of the Albany county Medical Society, on Erysipelas is instructive. The writer agrees with the views we have expressed on the relationship of erysipelas and puerperal fever, in our notice of Dr. Churchill's work on puerperal fever.

An essay by Dr. Wm. D. Purple: "On the Morbid Condition of the Generative Organs," is full of instruction. We shall recur to this subject in a future number.

There are several papers on cholera, among these proceedings, but we were disappointed in reading them. They are not what we should have looked for in a body possessing the talent that this State society does.

The State of New York publishes these proceedings annually as a part of the State documents, and is thus performing a portion of its duty to the people. We should like to see the Western and Southern States following the example of New York, and there is no portion of the Union that needs such information as is contained in this volume, more than the West and South. We should rejoice to see their mighty empires resting on a secure foundation of Hygiology.

